# Biased Signaling and Allosteric Modulation at the FSHR

**DOI:** 10.3389/fendo.2019.00148

**Published:** 2019-03-13

**Authors:** Flavie Landomiel, Francesco De Pascali, Pauline Raynaud, Frédéric Jean-Alphonse, Romain Yvinec, Lucie P. Pellissier, Véronique Bozon, Gilles Bruneau, Pascale Crépieux, Anne Poupon, Eric Reiter

**Affiliations:** PRC, INRA, CNRS, IFCE, Université de Tours, Nouzilly, France

**Keywords:** GPCR, reproduction, follicle-stimulating hormone, β-arrestin, G protein, signaling, bias, trafficking

## Abstract

Knowledge on G protein-coupled receptor (GPCRs) structure and mechanism of activation has profoundly evolved over the past years. The way drugs targeting this family of receptors are discovered and used has also changed. Ligands appear to bind a growing number of GPCRs in a competitive or allosteric manner to elicit balanced signaling or biased signaling (i.e., differential efficacy in activating or inhibiting selective signaling pathway(s) compared to the reference ligand). These novel concepts and developments transform our understanding of the follicle-stimulating hormone (FSH) receptor (FSHR) biology and the way it could be pharmacologically modulated in the future. The FSHR is expressed in somatic cells of the gonads and plays a major role in reproduction. When compared to classical GPCRs, the FSHR exhibits intrinsic peculiarities, such as a very large NH2-terminal extracellular domain that binds a naturally heterogeneous, large heterodimeric glycoprotein, namely FSH. Once activated, the FSHR couples to Gαs and, in some instances, to other Gα subunits. G protein-coupled receptor kinases and β-arrestins are also recruited to this receptor and account for its desensitization, trafficking, and intracellular signaling. Different classes of pharmacological tools capable of biasing FSHR signaling have been reported and open promising prospects both in basic research and for therapeutic applications. Here we provide an updated review of the most salient peculiarities of FSHR signaling and its selective modulation.

## Introduction

Follicle stimulating hormone (FSH) plays a crucial role in the control of male and female reproduction. FSH is a heterodimeric glycoprotein consisting of an α-subunit non-covalently associated with a β-subunit. The α-subunit is shared with luteinizing hormone (LH), chorionic gonadotropin (CG) and thyroid-stimulating hormone (TSH), whereas the β chain is specific of each glycoprotein hormone (1). FSH is synthesized and secreted by the pituitary and binds to a plasma membrane receptor (FSHR) that belongs to the class A of the G protein-coupled receptor (GPCR) superfamily. The FSHR displays a high degree of tissue specificity as it is expressed in Sertoli and granulosa cells located in the male and female gonads, respectively (2). FSH is required for normal growth and maturation of ovarian follicles in women and for normal spermatogenesis in men (3). Female mice with FSHβ or FSHR gene knockout present an incomplete follicle development leading to infertility, whereas males display oligozoospermia and subfertility (4, 5). Consistently, women expressing non-functional variants of the FSHR are infertile while men are oligozoospermic, yet fertile (6). To date, only native forms of FSH, either purified from urine or by using recombinant technology, are being used in reproductive medicine with no other pharmacological agents being currently available in clinic (7–9). Novel classes of FSHR agonists with varying pharmacological profiles could potentially help improving the overall efficiency of assisted reproductive technology. On the other hand, FSHR antagonists could represent an avenue for non-steroidal approach to contraception (10). This paper offers an updated overview of the way FSHR signals and of how selective modulation of its signaling can be achieved.

## Structure of the FSHR

**Figure 1:**
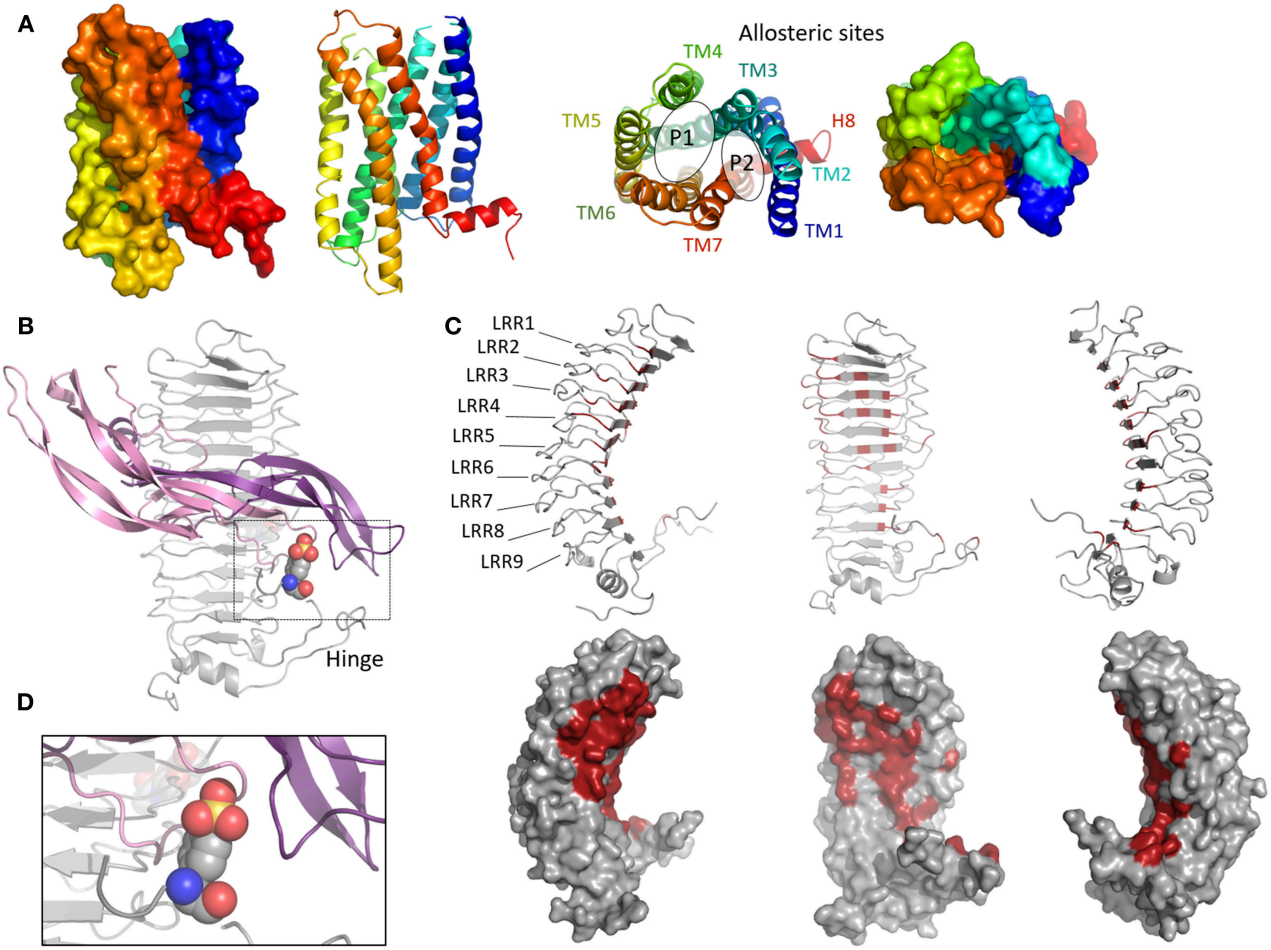
Orthosteric and allosteric sites in the FSHR. **(A)** Cartoon and surface view of the transmembrane regions of the FSHR showing P1 and P2 allosteric sites. **(B)** Complex between the ectodomain of the FSHR (gray) and FSH (violet: alpha chain, pink: beta chain). The colored spheres represent sulphated Tyr355. **(C)** Residues involved in FSH binding are shown in red. **(D)** Close-up on the interaction between sulphated Tyr335 (colored spheres) and FSH.

For the vast majority of GPCRs, the orthosteric site (i.e., the region that binds the natural ligand), is located in a cavity defined by the transmembrane helices. This is not the case for the FSHR, which binds its natural ligand, FSH, through its characteristic large horse-shoe-shaped extracellular domain (ECD). Consequently, the orthosteric site spans over the nine leucine rich repeats (LRRs) of the ECD (Figure 1). The first crystal structure of FSH bound to part of the ECD came out in 2005 and led to a detailed understanding of the molecular basis leading to the specificity of hormone binding (11). The ECD of the receptor contains 12 LRRs linked to three disulfide bonds and two unstructured sequence motifs that define the hinge region connecting the ECD to transmembrane domains (TMD). However, the recombinant protein used for crystallization did not include amino acid residues in the hinge region. Therefore, the receptor activation mechanism remained poorly understood, until recently, when another crystal structure including the hinge region was reported (12). Interestingly, it revealed a two-step activation mechanism of the receptor: interaction of FSH with LRR1-9 reshapes hormone conformation, so that exposed residues located at the interface of the hormone α- and β-subunits form a binding pocket for sulfated Tyr335 of the hinge region, resulting in a conformational change of the latter (13, 14). This two-step interaction process not only stabilizes the FSH/FSHR interaction but also relieves the tethered inverse agonistic activity previously mapped within the hinge region (15). Since no structural data of gonadotropin receptor TMD are currently available, homology modeling with other GPCRs is necessary. The structure of human neuropeptide Y1 receptor that recently came out (PDB:5BZQ) displays the highest identity with FSHR (25%) and LHR (24%) TM domains. Prior to that, gonadotropin receptor TM domains have been successfully modeled using adenosine receptor crystal structure (16). This revealed the existence of two adjacent pockets that could accommodate small ligands. These sites have been assigned P1 and P2 (major and minor site, respectively). The P1 site is located between TMs III, IV, V, and VI, and P2 between TMs I, II, III, and VII (Figure 1A) (17). These putative TM domain allosteric sites have been confirmed in studies utilizing chimeric receptors and mutagenesis. Interestingly, it was found that a FSHR small-molecule agonist at high concentration specifically displaced the binding of radiolabelled adenosine A3 receptor (A3R) agonist on A3R (18). This suggests a similarity between glycoprotein receptor and A3R in the TMD region for the allosteric binding pocket (19). As suggested from studies on other GPCRs, allosteric sites distinct from P1 and P2 may also exist and affect FSHR activity (20).

## FSHR Coupling to G Proteins

By analogy with other GPCRs, it is reasonable to posit that FSH binding leads to conformational rearrangements within the transmembrane regions, thereby causing the recruitment and coupling of signal transducers (G proteins, β-arrestins) that ultimately trigger a complex intracellular signaling network (21, 22). The primary transduction effector described for the FSHR is Gαs that triggers the canonical adenylyl cyclase/cAMP/protein kinase A (PKA) signaling cascade. Once activated, PKA phosphorylates many proteins such as transcription factors of the cAMP response element-binding protein (CREB) family (23–31). cAMP action is also mediated by the Exchange Proteins directly Activated by cAMP (EPACs) (32–34). Upon cAMP binding, EPAC1/2 stimulate Ras-related protein (RAP1/2), small GTPases that lead to protein kinase B phosphorylation (PKB) (35, 36). In addition, the FSHR has been reported to interact with Gαi and Gαq. Gαi inhibits adenylate cyclase, blocking Gαs-induced cAMP production (37). The stimulation of Gαq requires *in vitro* stimulation with high FSH concentrations (>50 nM) (22, 38–40). This coupling leads to the production of inositol 1,4,5 triphosphate (IP3) and diacylglycerol (DAG), increased intracellular calcium concentration and activation of protein kinase C (PKC). Pleiotropic coupling of FSHR to various heterotrimeric proteins suggests the co-existence of multiple active conformations of the receptor in the plasma membrane (41, 42).

## FSHR Coupling to β-arrestin

Similarly to most GPCRs, the FSHR interacts with β-arrestins, scaffolding proteins that control receptor desensitization, internalization and recycling (24, 43–46). Classically, β-arrestins are recruited following (i) receptor activation and (ii) receptor phosphorylation by G protein-coupled receptor kinases (GRK). Due to steric hindrance, FSHR coupling to Gαs is impaired once β-arrestins are recruited (47, 48). In a model of rat primary Sertoli cells that express the FSHR endogenously, it has been demonstrated that agonist-induced cAMP levels decreased upon β-arrestin overexpression, consistently with its role in FSHR desensitization (49). In heterologous cells, the carboxyl tail of FSHR has been reported to be phosphorylated on several serine and threonine residues (43). In addition to these classical functions, it has become increasingly clear that β-arrestins can also initiate specific, G protein-independent signaling events leading to the activation of many pathways, amongst which the ERK (Extracellular signal-Regulated Kinase) MAP (Mitogen-Activated Protein) kinase pathway has been the most studied (50). Of note, ERK activation kinetics at the FSHR has been reported to vary in heterologous cells as a function of the upstream transduction mechanism involved: β-arrestin-mediated ERK activation is delayed but more sustained compared to Gαs-dependent ERK activation, which occurs early but is transient (43). Consistent with the concept of “phosphorylation barcode” which links particular GRK-mediated phosphorylation signatures at the receptor level to the activation of distinct β-arrestin-dependent functions (51, 52), a relationship has been found between the subtype of GRK involved in FSHR phosphorylation and the nature of β-arrestin-mediated actions. In particular, β-arrestins recruited to GRK2 or GRK3-phosphorylated FSHR favor receptor desensitization whereas GRK5 or GRK6-mediated phosphorylation of FSHR were involved in β-arrestin-dependent ERK activation (43, 53, 54). Recently, phosphorylation of Tyrosine383 in β-arrestin 2 has proved to be crucial for β-arrestin-mediated ERK activation by the FSHR and other GPCRs. More precisely, ligand-induced receptor activation provokes MEK (Mitogen-activated protein kinase kinase)-mediated phosphorylation of Tyr^383^, necessary for β-arrestin 2-mediated ERK recruitment and activation (55). β-arrestins also play a role in FSHR-induced translation, mediated by a β-arrestin/p70S6K/ribosomal S6 complex that assembles in heterologous and in primary Sertoli cells. Upon FSH stimulation, activation of G protein-dependent signaling enhances p70S6K activity within the β-arrestin/p70S6K/rpS6 preassembled complex, leading to the fast and robust translation of 5′ oligopyrimidine track (5′TOP) mRNA (56). In addition, the balance between FSHR-mediated proliferation vs apoptosis seems to be regulated by β-arrestins. In hGL5 human granulosa cells, silencing of β-arrestins leads to an increase in cAMP/PKA and a decrease in β-arrestin-mediated proliferative pathway, resulting in cell death (57). Evidence reported for other GPCRs demonstrated that the internalized receptor can form molecular complexes involving simultaneous interactions with Gαs to the core domain and β-arrestin to the C-tail of the receptor (58). These complexes, named “megaplexes,” are able to signal from the endosome by inducing a second wave of cAMP (58, 59). Based on structural evidence, a two-step mechanism for β-arrestin recruitment has been proposed (60). First, β-arrestins are recruited to the phosphorylated C-tail, resulting in a so-called “partially engaged” complex which the authors reported to be sufficient for ERK signaling and internalization. Interestingly, this conformation allows the receptor to simultaneously couple to G protein α subunit. Second, a conformational rearrangement of β-arrestins allows them to interact with the receptor core domain, forming a “fully engaged complex” incompatible with further G protein coupling (58, 60–62). More recently, a separate study uncovered another mechanism of β-arrestin activation that the authors called “catalytic activation.” Upon ligand-induced recruitment of inactivated β-arrestin to the receptor core domain, a conformational change in β-arrestin occurs that exposes a PIP2-binding motif and allows β-arrestin to bind membrane lipid rafts independently of the receptor. Interestingly, the authors noticed an accumulation of active β-arrestin in clathrin-coated endocytic structures in the absence of the receptor, revealing the existence of a receptor C-tail-independent β-arrestin activation mechanism (63). No evidence currently exist that the aforementioned mechanisms also apply to the FSHR. Further studies will be necessary to clarify the molecular mechanisms involved in β-arrestin recruitment and activation at the FSHR and to determine their possible peculiarities.

## FSHR Interaction With Other Partners

Beside G proteins, GRKs and β-arrestins, the signal is also transduced at the FSHR by other direct binding partners (44). For example, adaptor protein, phosphotyrosine interacting with the Adapter protein with PH domain, PTB domain, and leucine zipper 1 (APPL1) binds intracellular loop 1 of the FSHR (64). This protein has lately retained the greatest attention in the gonadotropin community for two main reasons. The first one is that this adapter protein links the FSHR directly to inositide phosphate metabolism and Ca^2+^ release in granulosa cells (65), hence it induces cAMP-independent signaling; the second is that, like β-arrestins, APPL1 recruitment plays a role in the subcellular routing of FSHR. This discovery had been heralded by the previously identified interaction between GAIP-interacting protein C-tail (GIPC) adaptor and the FSHR (or the LHR), presumably requiring the carboxyterminal end of the receptor. Interestingly, GIPC reroutes the internalized FSHR from Early Endosomes (EE) to recycling Very Early Endosomes (VEE), and by these means, enables sustained ERK phosphorylation (66). Likewise, in HEK293 cells, APPL1 has been shown to convey internalized FSHR, as well as LHR, to VEE for recycling, and PKA-dependent phosphorylation of APPL1 leads to endosomal cAMP signaling (67). These two sets of observations on ERK MAP kinases and cAMP suggest that spatially restricted FSH signaling may be generalized to several of its components. In addition, 14-3-3τ has been shown to interact directly with the second intracellular loop of the receptor FSHR (68, 69). The 14-3-3τ interaction site on the FSHR encompasses the ERW motif involved in G protein association (70), that is consistent with the observation that 14-3-3τ overexpression in HEK293 cells reduces FSH-induced cAMP response (68). The co-occurrence of these direct binding partners as well as G protein, GRK and β-arrestins, raises fundamental questions about their sequence/dynamics of interaction on a single FSHR or about the possibility that FSHR oligomers might cluster transduction assembly of variable composition at the plasma membrane and in intracellular compartments.

## Trafficking and Endosomal Signaling

**Figure 2:**
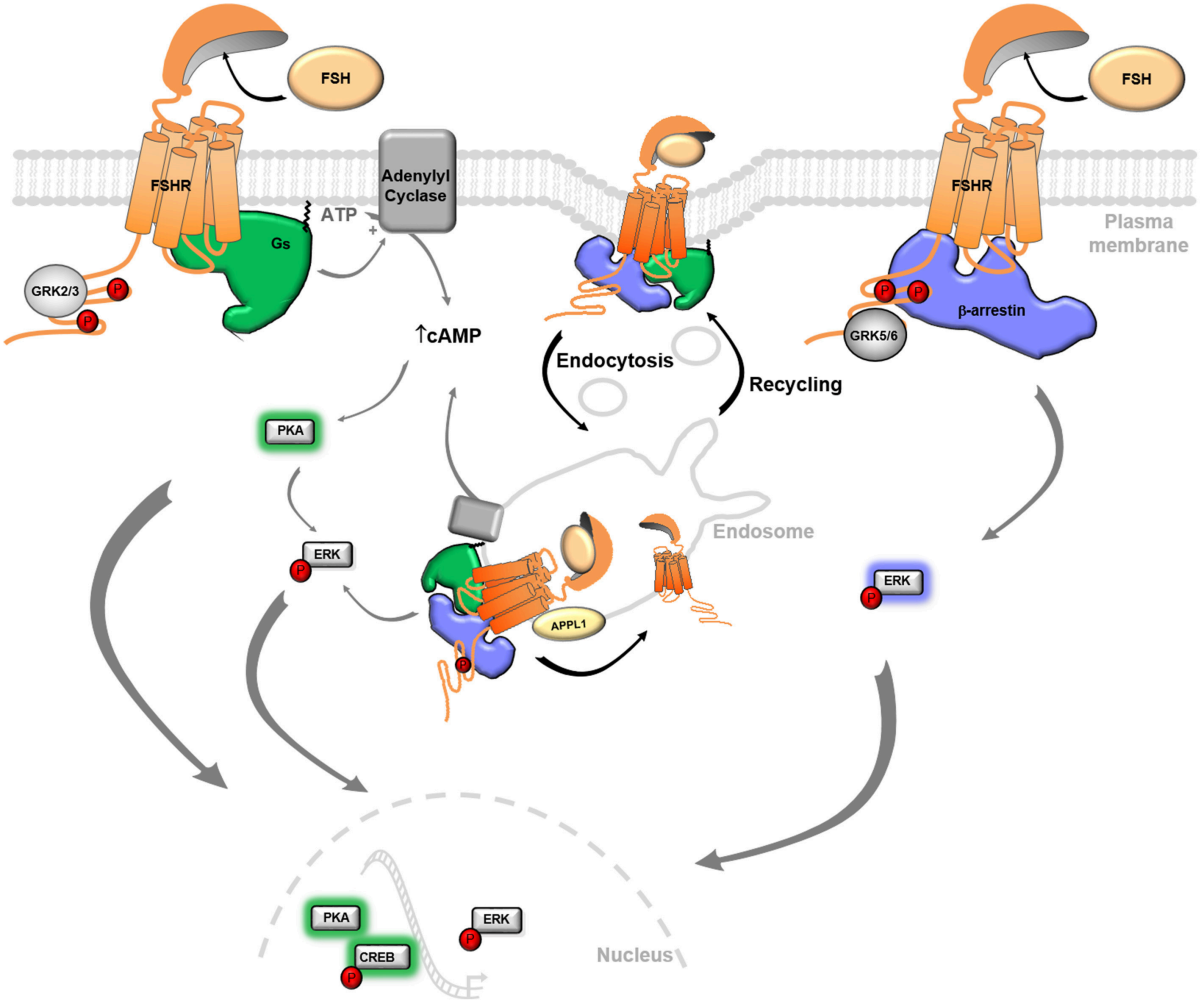
FSHR signaling and trafficking. Upon FSH binding, the FSHR mainly activates Gαs protein, leading to conversion of ATP to cAMP by adenylyl cyclases and activation of intracellular effector kinases, including PKA. After stimulation, GRK phosphorylates and desensitizes the FSHR. Phosphorylated FSHR recruits β-arrestin, which in turn induces its own signaling, including ERK activation, as well as receptor internalization in the endosomes. FSHR potentially activates G protein-dependent and -independent signaling from the endosomal compartment, before quickly recycling back to the plasma membrane. Effector proteins drive the cellular responses, including gene transcription, cell proliferation and differentiation. APPL1, Adaptor protein, phosphotyrosine interacting with PH domain and leucine zipper 1; CREB, cAMP response element binding protein; ERK, extracellular signal-regulated kinase; FSH, Follicle-stimulating hormone; GRK, G protein-coupled receptor kinase; PKA, protein Kinase A.

Compartmentalization of signaling is now viewed as an important feature for many signaling proteins and plays key roles in cellular responses. This is particularly the case for membrane receptors such as GPCRs since, in the past years, several examples have revealed connections between membrane compartmentalization, endocytic trafficking and signaling patterns. Originally thought to function solely at the plasma membrane, the multifunctional protein β-arrestin assemble signaling molecules (e.g., MAPK, Src, etc) that direct GPCRs to the endocytic pathway and regulate their post-endocytic fate, as mentioned above. For some GPCRs forming a stable interaction with β-arrestin, β-arrestin/receptor/signaling molecule complexes are found in endosomes, allowing prolonged signaling from these intracellular structures (71–73). The nature of the β-arrestin binding motifs, in particular serine/threonine clusters in the C-tail of the receptor, regulates the stability of this interaction. GPCRs that display high affinity binding to β-arrestin, are classified as class B (74, 75). In the FSHR, a cluster of 5 serines/threonines is involved in both internalization and binding of β-arrestin to the receptor and is consistent with the class B definition. Such interaction was confirmed by bioluminescence energy transfer experiments (BRET) and co-immunoprecipitation experiments, however no imaging data have confirmed the existence of a functional complex in endosomes (24, 43). In addition, it is unclear whether β-arrestin-mediated ERK signaling by FSHR requires β-arrestin localization in endosomes. Recently, both the FSHR and LHR were reported to predominantly localize to an atypical endosome denoted as VEE (Figure 2). Alteration of this endosomal trafficking by blocking internalization inhibits activation of ERK through the LHR, suggesting that VEE are a location for signaling (66). These particular endosomes are upstream of the classical endosomes and are devoid of typical early endosomes markers such as the Rab5 GTPase or the phosphatidylinositol-3-phosphate (PI(3)P) or the PI(3)P-bound EEA1 proteins. Morphologically, they are smaller (<400 nm) than conventional sorting EE but their exact nature remains to be defined. Interestingly, gonadotropin receptor localization in VEE requires an intact receptor C-tail and the GIPC PDZ-domain protein (66). PDZ motifs are found in several GPCRs to regulate their spatial localization or trafficking (76). The PDZ motif of the LHR directly binds GIPC that sequesters the receptor into VEE following agonist-induced internalization. In fact, in cells depleted in GIPC or expressing LHR lacking the distal PDZ motif in its C-tail, the receptors are rerouted and accumulated into the classical EE where they fail to recycle back to the plasma membrane. In addition, they were not able to signal to ERK MAP kinases (66), suggesting that endosomal ERK activity occurs from this specific compartment. It is worth noting that the FSHR does not display a known PDZ ligand in its C-tail and the exact mechanism on how GIPC controls FSHR fate remains to be determined. However, APPL1, a known FSHR binding partner, localizes to a subset of VEE and displays a PDZ motif previously shown to interact with GIPC (64, 65, 77). A possible scenario would be that FSHR, *via* its interaction with APPL1, connects with GIPC and is targeted to VEE where it activates ERK (78). Earlier work supports the idea that endosomal APPL1 defines a signaling platform upstream of the Rab5/PI(3)P endosomes. Disruption of EE leads to the accumulation of the EGFR Tyrosine kinase receptor in APPL1 vesicles, leading to a sustained activation of ERK from this compartment (79, 80). As shown for the LHR, the endosomal cAMP/PKA dependent phosphorylation of APPL1 on Ser410 is necessary for the recycling of the receptor (67).

This concept of endosomal signaling compartmentalization was further supported by the findings that GPCR can induce a second phase of G protein activation following their internalization (81–83). This allowed the advent of a new paradigm where some GPCRs do not only transduce and activate G proteins from the plasma membrane but also from endocytic compartments (the so-called “megaplex” mentioned above). Interestingly, the two other members of the glycoprotein hormone receptor family, TSHR and LHR, were both shown to transduce *via* Gαs and promote sustained cAMP production from endocytic compartments (59, 67, 84, 85). While the TSHR acts from EE and *trans*-Golgi compartments, the LHR signaling is restricted to the VEE. It has yet to be shown whether the FSHR also activates G proteins from the VEE but the fact that it shares several features with GPCRs known to signal from endosomes, including LHR, V2R or PTHR, supports this possibility. FSHR trafficking mimics the LHR as discussed above and it displays a phosphorylation code similar to those found in the C-tail of V2R and PTHR. More precisely, the formation of a “megaplex” has been shown to induce cAMP from the EE in response to PTHR activation (86, 87). That the FSHR could signal in endocytic compartments through G proteins in a similar way as the PTHR, but from VEE, is conceivable, but further studies are needed to demonstrate this possibility. Despite the identification of structural determinants in the FSHR C-tail that regulate its trafficking (88), very little is known about the mechanisms involved in the post-endocytic trafficking of this receptor.

## Biased Signaling and Its Quantification

The action of a given ligand on its cognate receptor has classically been characterized by its effect on downstream effectors (second messengers). Compared to the reference ligand (generally, the physiological ligand), a pharmacological agent can be either an agonist (it produces a biological response similar to that of the reference ligand), an antagonist (it blocks the biological response elicited by the reference ligand) or an inverse agonist (it produces an opposite biological response that leads to a decrease in the receptor basal activity). Importantly, it has long been thought that these characteristics hold irrespectively of effector measured (89). However, some ligands did not match with one of these categories, because they displayed both agonist and antagonist (or inverse agonist) activities at the same receptor, depending on the downstream pathway measured. For instance, carvedilol, a clinically used β-blocker, has a clear inverse agonist profile on Gαs-dependent activity at the β2 adrenergic receptor while being a weak partial agonist for β-arrestin-dependent ERK activation (90). To deal with these discrepancies, the concept of biased signaling or functional selectivity, recently came to the fore (89, 91, 92). According to this concept, a ligand is biased when it triggers imbalanced responses, compared to a reference ligand acting on the same receptor (classically the endogenous ligand). Importantly, a biased ligand can selectively activate only a subset of the biological responses or activate all of them but with different efficacies compared to the reference ligand. As these ideas hold profound implications and potential for the design of new therapeutics, biased signaling is a very active area of research in pharmacology (93, 94). Over the last decade, biased signaling has been evidenced in many different receptors, including gonadotropin receptors, as will be discussed in a forthcoming section. By analogy with a ligand bias, the notion of receptor bias has been proposed (95). Two receptors, diverging only by a mutation or a polymorphism, once activated by the same ligand may induce two signaling pathways with different relative efficacies. Importantly, biased signaling has been extended to allosteric ligands, which can modulate not only the efficacy of a given ligand-induced receptor signal but also select and bias the activation of the receptor toward a subset of the biological responses (93). Ligand bias and receptor bias must be set apart from system bias or observational bias (95). System bias refers to bias that are due to the particular biological system used (some transducer molecules, such as G proteins, may be expressed differentially in different cell types for instance). Observational bias refers to the modification or amplification of the signals inherent to the specific assays used for the measurements. Besides being supported by numerous experimental evidences, biased signaling is consistent with the receptor conformation theory, which views a receptor population as an ensemble of conformations that evolve dynamically, according to some energy landscape and subjected to external perturbations (96). In such theory, ligand-induced receptor activation is concomitant with a stabilization of some receptor conformations and a modification of the receptor conformation energy landscape, resulting from the interaction of the ligand with its receptor. Several studies have thus shown that receptor conformational equilibrium models, such as the extended ternary complex model, can satisfactorily explain ligand bias (97, 98). Several groups have attempted to address the problem of bias quantification. Considering a given receptor and two signaling pathways (A and B), the objective is to be able to classify ligand bias, as having for instance a low, a moderate or a high bias toward pathway A vs pathway B, when compared to a reference ligand. The most popular method to quantify ligand bias uses dose-response data and the so-called operational model (99, 100). The latter is widely used to perform regression on dose-response data, which have in many cases a sigmoid shape. The parameters of the operational model are derived from a simple chemical reaction scheme that takes into account ligand receptor association/dissociation reactions and that links the ligand-receptor concentration to the biological response thanks to a logistic function (similar to enzymatic reaction models). The usefulness of this model for the quantification of ligand bias is associated with the interpretation of its parameters. In particular, the two main parameters of the operational model are the ligand-receptor dissociation constant K_a_ and the intrinsic efficacy τ (which describes the ability of the ligand-receptor association to be converted into a response). With these two coefficients, a single transduction coefficient, given by log(τ/K_a_), has been proposed to characterize the agonism of a ligand for a given signaling pathway (100). This coefficient can then be compared between two pathways and between two ligands, to ensure normalization. Hence

$$\begin{array}{l} \Delta \text{log}\left(\tau /{\text{K}}_{\text{a}}\right)=\text{log}{\left(\tau /{\text{K}}_{\text{a}}\right)}_{\text{ligand A}}-\text{log}{\left(\tau /{\text{K}}_{\text{a}}\right)}_{\text{ligand B}} \end{array}$$

quantifies the activation of a pathway by ligand A, compared to ligand B. In addition,

$$\begin{array}{l} \Delta \Delta \text{log}\left(\tau /{\text{K}}_{\text{a}}\right)=\Delta \text{log}{\left(\tau /{\text{K}}_{\text{a}}\right)}_{\text{pathway }1}-\Delta \text{log}{\left(\tau /{\text{K}}_{\text{a}}\right)}_{\text{pathway }2} \end{array}$$

evaluates the differences of activation between the two pathways. Finally, the bias is defined as

$$\begin{array}{l} \text{bias}=\;10^{\text{Δ}\Delta \text{log}\left(\tau /\text{Ka}\right)} \end{array}$$

This procedure for bias quantification, together with its statistical significance, has been detailed as a step-by-step protocol using Prism (v6.0; GraphPad Software) (101–103). Other logistic regressions that lead to different quantifications and parameter interpretations have been proposed and compared (104). A notion of dose-dependent ligand bias, which may reveal subtle nonlinear effects of the ligand, has also been defined using logistic function (105). Overall, the statistical regression of dose-response data (sigmoid curves) can be ambiguous and lead to a misinterpretation of the results. Moreover, it has been shown experimentally that different procedures may exhibit discrepancies between each other, and may fail to detect ligand bias or lead to false positives, probably due to the presence of system or observational bias (104). A semi-quantitative method to classify ligand bias that would be more robust than quantitative methods has been proposed, based on logistic regression (104). However, the major concern of the operational approach to quantify ligand bias is that it disregards an important aspect of signaling pathways, namely the temporal activation of the different signaling processes (106). This has been revealed by the relatively simple observation that the bias value, as calculated with the operational model, could change as a function of the kinetics of response (107). Actually, the apparent bias can even be in an opposite direction for two different time points when the biological responses are measured. While part of the explanation of this phenomena resides in the different time scales at stake within a signal transduction pathway (binding kinetics, second messenger and effector kinetics), it also reveals the whole complexity of a receptor trafficking system (108), that can certainly not be condensed into a single number. Thus, methodological developments such as dynamical versions of the operational model and/or the extended ternary complex model (109, 110) must be developed to address this complexity and allow better characterization of the effect of a ligand on its cognate receptor.

## Biased Signaling at the FSHR

**Figure 3:**
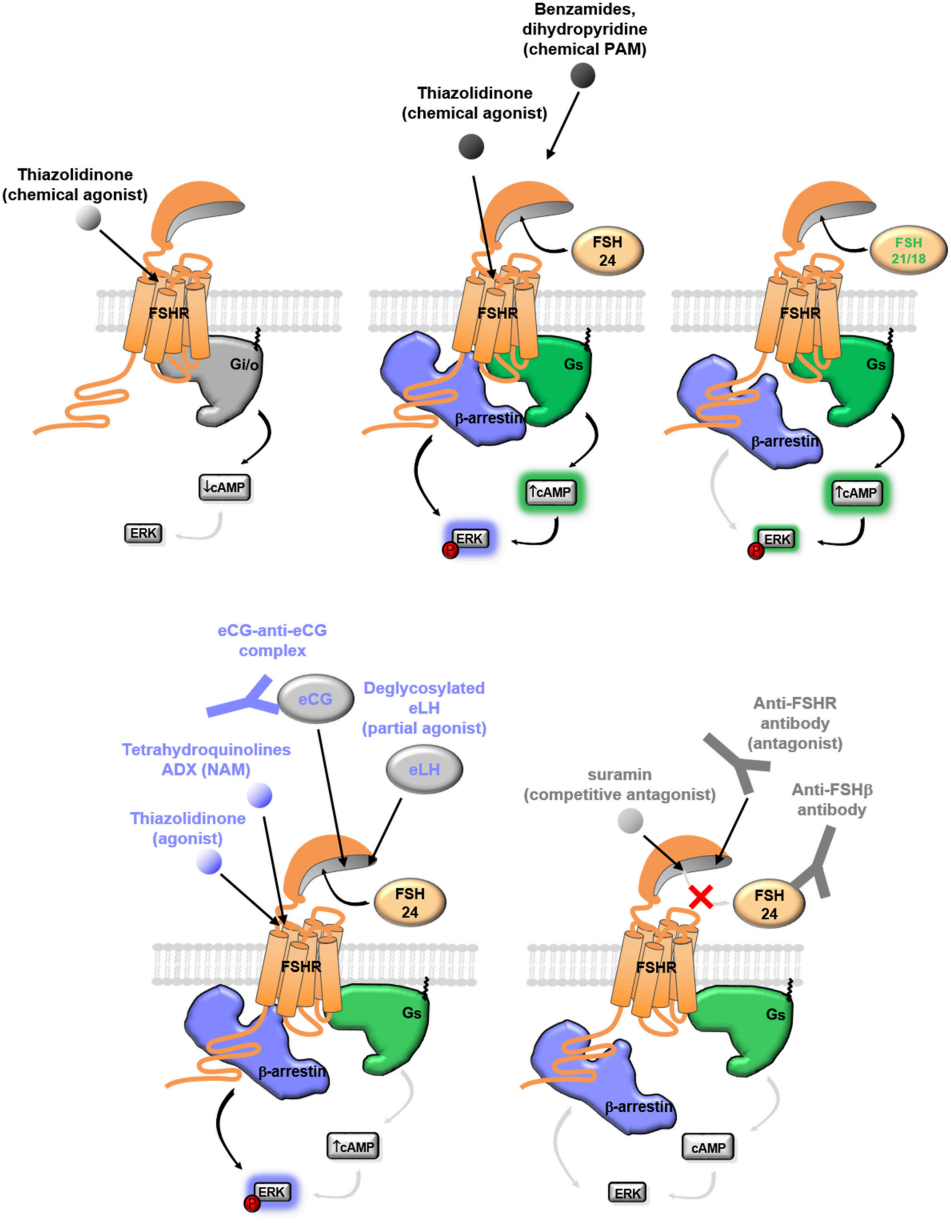
Ligand bias at the FSHR. Balanced agonists or PAM at the FSHR induce both G protein and β-arrestin recruitment. FSH binding to the FSHR can be prevented using small competitive ligands, antibodies directed against the binding pocket of the FSHR or directly against FSH. Biased signaling toward Gαi, Gαs, or β-arrestin recruitment can result from glycosylation forms of FSH (fully glycosylated FSH24 vs partially glycosylated FSH21-18), antibody or small chemical compounds.

To date, different classes of biases have been reported to elicit selective modulation at the FSHR (Figure 3). Ligand bias can be provoked by small molecule ligands, glycosylation variants of FSH or by antibodies acting at FSH or FSHR. Receptor bias due to mutations or single nucleotide polymorphisms (SNP) at the FSHR have also been reported.

### Small Molecule Ligands

Several classes of chemical compounds exhibiting the ability to modulate FSHR-mediated signaling upon binding have been identified to date. Readers interested in the chemical diversity of currently known FSHR small molecules classes can refer to Figure 2 of Anderson et al. in the same special issue of Frontiers in Endocrinology (19). According to their mode of action and effect on the receptor, they can be divided in four classes: allosteric agonists, positive allosteric modulators (PAMs), negative allosteric modulators (NAMs) and neutral allosteric ligands (NALs) (111). While PAMs or NAMs need the presence of FSH to detect the enhancement or the decrease of FSHR activation, respectively, agonists have the capacity to activate it on their own. Even though NALs do not influence signaling, they can potentially prevent other allosteric modulators from binding (112). Thiazolidinones, identified by screening combinatorial chemical scaffolds, were the first class of FSHR allosteric agonists to be reported (113). The allosteric nature of thiazolidinone derivatives was confirmed thanks to experiments involving FSHR/TSHR chimeras, which showed that their binding site was localized in the TMD (114). A nanomolar potent thiazolidinone FSHR agonist was reported to trigger signaling pathways similar to FSH, both *in vitro* and *in vivo* (115). Interestingly, some thiazolidinone analogs demonstrated biased agonism by mobilizing the Gαi protein instead of Gαs or both as observed for other thiazolidinone analogs or FSH preparations (116). Besides, high throughput screening on substituted benzamides allowed the identification of a series of FSHR PAMs that showed improved selectivity against LHR and TSHR. Interesting pharmacokinetic properties were also described for two selected compounds (117). A dihydropyridine compound, Org 24444-0, is another PAM, which displayed a good selectivity toward FSHR and induced cAMP production in presence of FSH (118). The compound was also able to reproduce the effects of FSH on the follicle phase maturation in mature female rats. Among the currently known NAMs, tetrahydroquinolines constitute a good example of biased signaling. It was indeed established that the compounds inhibited FSHR-induced cAMP production, without inhibiting FSH binding (119). Unfortunately, the tetrahydroquinolines did not display any *in vivo* activity. Three other NAMs have been characterized by Dias et al. (120, 121). The first one, ADX61623, was reported to inhibit cAMP and progesterone but not estradiol production in rat granulosa primary cells. Using ^125^I-hFSH, it was established that ADX61623 did not compete with FSH, but rather increased FSH binding, suggesting that it does not bind the extracellular domain of FSHR. When tested *in vivo*, the compound was not able to decrease FSH-induced preovulatory follicle development (120). Two similar compounds were described later: ADX68692 and ADX68693. Both were reported to inhibit cAMP and progesterone production in rat granulosa primary cells, but while ADX68692 also affected estradiol and decreased the number of oocytes recovered in mature female rat, ADX68693 had no effect on estradiol, nor on the number of retrieved oocytes (121). Interestingly, ADX68692 and ADX68693 were also reported to exert similar actions on the LHR (122). The first FSHR competitive antagonist described in scientific literature, suramin, was reported to inhibit testosterone production and FSHR signaling, by competing with FSH binding (123). Another non-competitive antagonist of human and rat FSHR showing the same behavior was later identified (124).

### Glycosylation Variants

Gonadotropins present natural heterogeneity in their glycan moieties that contribute up to nearly 30% of the hormone's mass (125–128). The presence of glycans has important outcomes on the *in vivo* half-life of the hormone because, by doubling its diameter, it limits its glomerular filtration. FSH contains two potential N-linked oligosaccharides on each subunit that are sources of heterogeneities. Importantly, these glycan chains are involved in FSH folding, assembly, stability, quality control, secretion, transport as well as the biological activity and potency (15, 129–138). The α chain is glycosylated at asparagine 52 (Asn52) and Asn78, while the FSH β subunit can be glycosylated at Asn7 and Asn24. Partially glycosylated variants that are missing either one or both of these oligosaccharides on FSHβ have been reported in equine FSHβ, human FSHβ (hFSH β), rhesus FSHβ and Japanese macaque FSHβ (139–142). Glycosylation profile of each subunit plays a critical role in the activity and clearance of FSH (131, 143, 144). Interestingly, while FSHα subunit amino-acid sequences are identical to LH, TSH and CG α-subunits, the N-glycan populations at Asn52 and Asn78 differ from those of the other hormones (145–147). FSHβ subunit shares 34–40% of sequence homology with the other human glycoprotein hormone β-subunits, yet the main structural hallmarks (i.e., six disulfide bonds, cystine knot motif and seatbelt loop) are conserved (148, 149). Interestingly, the abundance of the glycosylated variants in FSHβ subunit appears to be physiologically regulated (141). Although glycosylations are involved in the FSH bioactivity, they are not directly interacting with the receptor binding site (11, 12, 15, 150). Removal of the carbohydrate residue at position 78 from α-subunit significantly increases receptor binding affinity of human FSH. Likewise, carbohydrate at position 52 of the α-subunit was found to be essential for bioactivity since its removal resulted in significant decrease in potency. Furthermore, β-subunit carbohydrates are essential for FSHβ/FSHα heterodimerization (138, 151). In binding assays, hypoglycosylated FSH (triglycosylated FSH21/18, missing either Asn7 or Asn24-linked oligosaccharide on the β chain) was 9–26-fold more active than fully glycosylated FSH (tetraglycosylated FSH24) (139). Likewise, a deglycosylated FSH variant, which possesses only α-subunit oligosaccharides, is significantly more bioactive *in vitro* and more efficient in receptor binding than the tetraglycosylated form of the hormone (141, 142, 152). However, this hypoglycosylated FSH is not physiologically relevant because subunit heterodimerization is extremely inefficient when both FSHβ glycans are missing, precluding secretion of enough active forms (151). In contrast, ovulated eggs and subsequent *in vitro* embryo development was increased by hyperglycosylated FSH (153). FSH variant abundance is tightly correlated with fertility: FSH24 predominates in men and post-menopausal women whereas FSH21/18 is more abundant in younger females. This observation suggests that hypoglycosylated FSH may play a preferential role in efficient stimulation of ovarian follicle development (154). Noteworthy, FSH variants have been reported to exhibit biased signaling: FSH21/18 is better to activate the cAMP/PKA pathway and is 10-fold more potent in inducing CYP19A1 and estrogen than fully glycosylated FSH24 (155). Bias at the FSHR has also been reported with partially deglycosylated eLH (eLHdg) preparation. β-arrestin depletion revealed that eLHdg induced β-arrestin recruitment to the FSHR and activated both ERK and PI3K pathways in a β-arrestin-dependent and Gαs/cAMP-independent manner (156). Altogether, these data suggest that FSH glycoforms may act as physiological bias (157). A recent study revealing signaling bias between human LH and hCG is consistent with this hypothesis (158).

### Antibody

Particular antibodies have been shown to selectively modulate FSHR activation, likely eliciting structural constraints and stabilizing distinct conformations of FSH and/or its receptor (21). Monoclonal antibodies against bovine FSHβ and anti-peptide antibodies targeting ovine FSHβ both significantly enhanced biological activity in mice (159, 160). Interestingly, in non-equine species, equine CG (eCG) binds to both FSHR and LHR and elicits their activation (161–164). Studies have evaluated the impact on gonadotropin bioactivities of different eCG/anti-eCG antibody complexes generated using individual sera from a large number of eCG-treated goats. Interestingly, both inhibition and hyperstimulation of LH and FSH bioactivity were recorded (165). In a follow-up study, Wehbi et al. investigated the effects of these complexes on FSH signaling in more details (166). Three stimulatory complexes were tested, displaying modulatory effect on cAMP production but all exhibited increased β-arrestin-dependent ERK response, suggesting biased properties. Recently, Ji et al. developed two anti-FSHβ monoclonal antibodies using synthetic peptides located at the binding interface of FSHR (167). Strikingly, this study demonstrated that blocking FSH action using antibodies against FSHβ protects ovariectomized mice against bone loss, by stimulating new bone formation and reducing bone removal besides inhibiting fat accumulation. Direct targeting of GPCR with antibody or antibody fragments in order to modulate their signaling is increasingly viewed as a viable approach that even led to therapeutic applications in the last few years (168). The FSHR has been targeted by antibodies in different studies. Recombinant filamentous phages displaying at their surface three overlapping N-terminal decapeptides of the FSHR, A18–27, B25–34, and C29–38 peptides were used for immunizing ewes and female mice. When tested *in vitro*, antiA and antiB immunoglobulins behaved as antagonists for FSH binding and for cAMP production, whereas antiC immunoglobulins did not compete for hormone binding but displayed agonist activity on FSHR-mediated cAMP response (169). Studies using polyclonal and monoclonal antibodies or scFv fragments specific of the hinge region of FSHR, LHR, or TSHR, while not affecting hormone binding, all revealed agonistic activities, unequivocally establishing the role of the hinge region in the activation of these receptors (170–172). More recently, recombinant nanobodies capable of specifically recognizing FSHR and of inhibiting cAMP accumulation have been identified (173). Even though the biased nature of the above-discussed anti-FSHR antibodies have not been assessed in the original studies, it is tempting to speculate that antibodies and antibody fragments hold a lot of promises as research tools and as therapeutic agents capable of eliciting functional selectivity at the FSHR.

### Single Nucleotide Polymorphisms and Mutations

**Figure 4:**
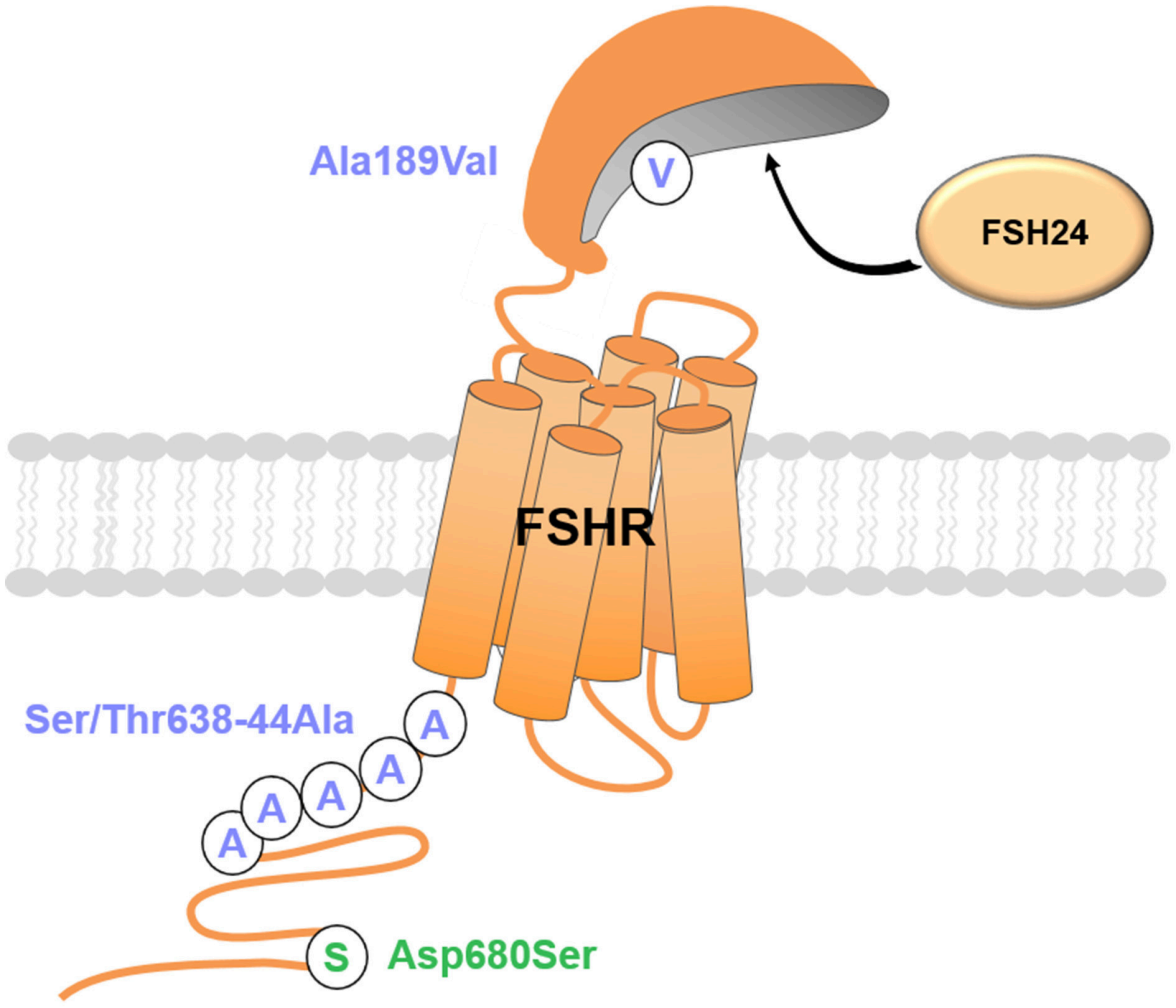
Mutation-induced receptor bias at the FSHR. Mutations can lead to biased signal transduction at the FSHR upon exposure to fully glycosylated FSH (FSH24). Green, Gs-biased mutants; purple, β-arrestin-biased mutant.

Induced or natural mutations have been shown to elicit biased signaling in various GPCRs (174–176). In the FSHR, active and inactive mutations and SNP have been reported (177) but most of them are insufficiently documented to suggest they could induce a receptor bias. However, some studies suggested that a mutation or a SNP at the FSHR can modify the balance between different signaling pathways (Figure 4). The Ala189Val inactive mutation, leading to subfertility in men and infertility in women, impairs the G protein pathway but not β-arrestin-dependent ERK activation (6, 178). However, this Ala189Val mutation provokes intracellular retention of the FSHR, hence decreases its plasma membrane expression level (179). Tranchant et al. demonstrated that the FSHR also elicits preferential β-arrestin-dependent signaling when its plasma membrane density is similar to that of the Ala189Val mutant. Therefore, the Ala189Val mutation could very well represent a case of system bias rather than of receptor bias. Uchida et al. described an inactivating mutation (Met512Ile) in the FSHR of a woman with ovarian hyperstimulation syndrome (OHSS) but probably not related with this pathology (179). The mutant receptor led to decreased cAMP and PI3K responses whereas ERK activation remained unchanged compared to wild-type FSHR. Further investigations are required to ascertain whether the imbalance between the different signaling pathways is caused by a true receptor bias or whether it also results from affected cell surface expression of the receptor. Another case is the Asp431Ile mutation in the extracellular loop 1 (EL1) that has been found in a man with undetectable circulating FSH but normal spermatogenesis (180). This mutation leads to a marked decrease in FSH-induced desensitization and internalization compared to the wild-type receptor.

The FSHR gene carries about 2,000 SNPs, among which the SNP p.N680S (c.2039A>G) is a discrete marker of ovarian response. Women bearing the serine variant display resistance to FSH compared with those bearing the asparagine variant. p.N680S S homozygous FSHR differently stimulates intracellular cAMP and leads to different kinetics of ERK and CREB phosphorylation (181). Kara et al. have shown that site-directed mutagenesis of all the five ser/thr residues located in the C-tail at position 638–644 of the rat FSHR reduced its ability to interact with β-arrestins upon FSH stimulation (43). Interestingly, the internalization of the mutant receptor was reduced while its ability to activate ERK via the β-arrestin-dependent pathway was increased, indicating receptor bias.

## Conclusions

The observation that FSHR transduction can be finely tuned by a variety of biased ligands, mutations or polymorphisms, further emphasizes the importance to better understand the complex signaling networks that are modulated (i.e., activated or inhibited) downstream of the FSHR. These novel biased ligands and receptor variants are great research tools that should really help us deciphering the molecular mechanisms involved in FSHR-associated physiopathology. In addition, a number of existing ligands and mutants have been characterized solely by measuring plasma membrane expression and/or cAMP response. Further characterization is required and may generate insightful findings. Biased ligands also open intriguing prospects in drug discovery. In particular, low molecular weight agonists of the FSHR could lead to the development of orally-active treatments. Such administration route would bypass the multiple injections of gonadotropin preparations that remain needed in the current protocols used in assisted reproduction. Moreover, it becomes possible to sort out the pathways leading to ovulation and those responsible for OHSS, and the availability of pathway-selective low molecular weight agonists at the FSHR could pave the way for the development of safer treatments, presenting reducing risks of OHSS. Modulation of relative FSH and LH activities could also open new avenues in the treatment of polycystic ovarian syndrome (PCOS).

On a more general note, the availability of allosteric compounds active at the FSHR, opens the unprecedented opportunity to enhance or dampen the transduction activities of the FSHR *in vivo*, while conserving the rhythmicity and biochemical diversity of endogenous FSH, a property that no orthosteric compound can match. The conditions of application of such treatments will obviously require extensive pre-clinical and clinical studies. Despite of these limitations, hampering any hope for short-term clinical use, the advent of biased and allosteric compounds certainly represents an important juncture in a field that has uniquely relied for so long on natural and recombinant gonadotropins to treat infertility. Finally, orally active low molecular weight FSHR antagonists may also lead to novel classes of oral contraceptives devoid of the side effects associated with current sex steroid-based contraceptives.

## Author Contributions

Each author wrote a section of the review. AP and LPP designed Figures 1–4 respectively. ER integrated all the contributions into the final manuscript. All authors edited the complete paper.

### Conflict of Interest Statement

The authors declare that the research was conducted in the absence of any commercial or financial relationships that could be construed as a potential conflict of interest.
